# The Prevention of Malaria

**Published:** 1900-03-24

**Authors:** 


### THE PREVENTION OF MALARIA.

If we consider the factors necessary for the continuance of malaria in any district it is clear that the chain of events on which its prevalence depends can be broken at several points. Granting that the malaria parasite passes one portion of its life in the blood of man, and another equally essential stage of its existence in the body of the mosquito, it is clear that if we could kill all the mosquitos of the district no man within it could catch malaria, while if we could render all the men of the district free from malaria no mosquitos could become infected with the parasite. The same results would also be obtained if men and mosquitos could be kept apart during the night, that is, during the time when the mutual transference of the infection occurs.

At a meeting held at the Colonial Institute last week Dr. Patrick Manson described some experiments which he has been authorised by the Colonial Office to make, so as to demonstrate the practicability of preventing malarial infection in intensely malarial localities by easily applied means. A hut is to be erected in the most malarial part of the Roman Campagna. It is to be furnished with wire gauze door and window screens, and in other ways is to be rendered mosquito proof. Two skilled observers and their servants are to live in this hut through the summer of this year; that is, through the whole malarial season. It is arranged that they are to go about as they like during the day, but that from one hour before sunset to one hour after sunrise they are to be confined to their mosquito-proof hut. Now, it is well recognised by the Romans that to sleep for a single night unprotected at the spot chosen is tantamount to contracting fever of a virulent type; and if the people shut up in this hut—open to the air, but shielded from the mosquitos by the wire netting—manage to escape the fever, it will be the strongest possible proof that by simple measures properly applied the human body can be protected from this deadly disease.

Another experiment which is to be made is as follows: Mosquitos raised from the egg in the laboratory, and therefore free from malaria germs, are to be fed in Rome on patients in whose blood the benign tertian malaria parasite has been proved by microscopical examination to be present, and then these infected mosquitos are to be brought to London, where they are to be fed on vegetable juices, that is, on non-malarial food, until time enough has elapsed for the malaria germs to have arrived at the venom gland of the mosquito, and thus be ready for being injected into the body of any man whom the creature may attack. These insects are then to be liberated into a mosquito house in which one or more malaria-free Englishmen are to sleep, and if in about ten days after this adventure these experimenters find themselves affected with malaria we shall again have proof of a somewhat striking character, of the part played by the mosquito in spreading the disease; a proof, it may be added, which now is hardly needed.

What is really wanted is a proof of what we think as yet is hardly proven, namely, that man's is the only blood in which the parasite will go through its blood stage of existence. This does not matter if one is to set to work to stop malaria by killing the mosquito—a large task; but it is now held that malaria could be stamped out in a district if one could but ensure, for a time, all-round free drugging with quinine, it being argued that after a month or so the supply of infected blood would have come to an end, and consequently there would be no more infected mosquitos. In small communities this might appear a far simpler plan than that of attacking the mosquitos, but evidently it would be doomed to failure if some other creature were all the time supplying the mosquitos with infected blood. Granting, as we all do, that the mosquito is the sole agent by which man is infected, the proof does not as yet seem equally clear that man is the sole agent from whom the mosquito gets its dose. The experiments which Dr. Manson is about to perform are interesting as demonstrations, and will no doubt bear good practical fruit, but if the Colonial Office could arrange for Dr. Manson to have the whole population of some small malaria-bitten locality thoroughly dosed with quinine for several months, and then see what happened to a malaria-free Englishman if he slept in the open, the experiment would be worth something.

